# Supplementary material for: The representation of colored objects in macaque color patches
Source: Nat Commun. 2017 Dec 12;8:2064. doi: 10.1038/s41467-017-01912-7 (PMC5727180; doi:10.1038/s41467-017-01912-7)
Supplement: Supplementary file 1 — Supplementary Information [file 41467_2017_1912_MOESM1_ESM.pdf]

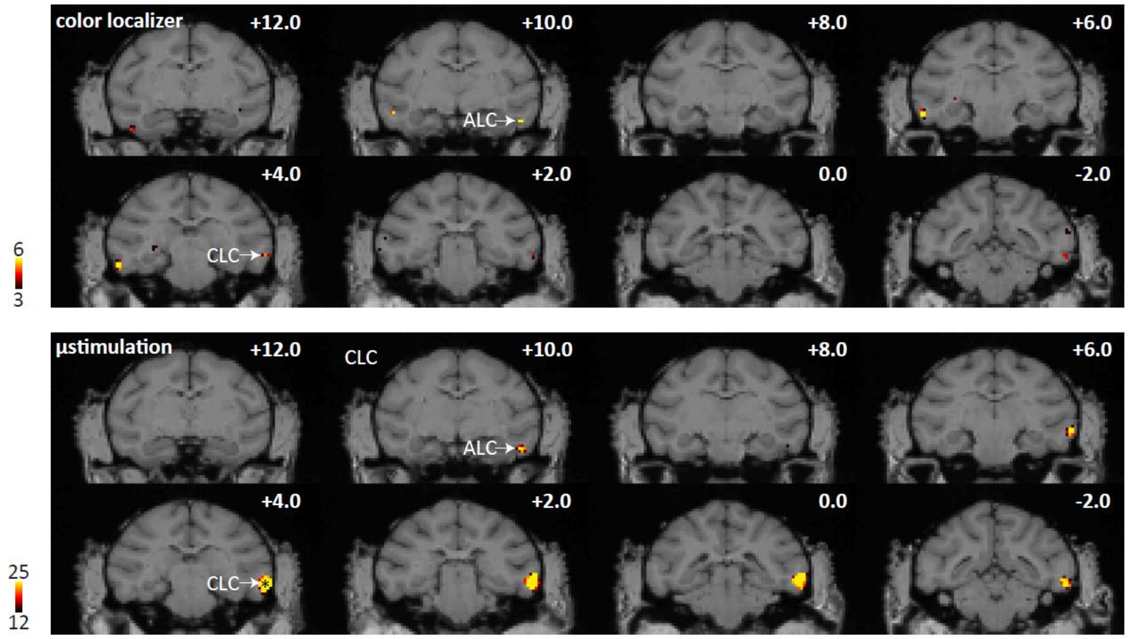

Supplementary Figure 1. Comparison between color localizer and activation by microstimulation in color patch CLC.

Same convention as Fig. 1d, but for microstimulation in color patch CLC in the right hemisphere of monkey M2 (* indicates stimulation site). Source: Tsao lab.

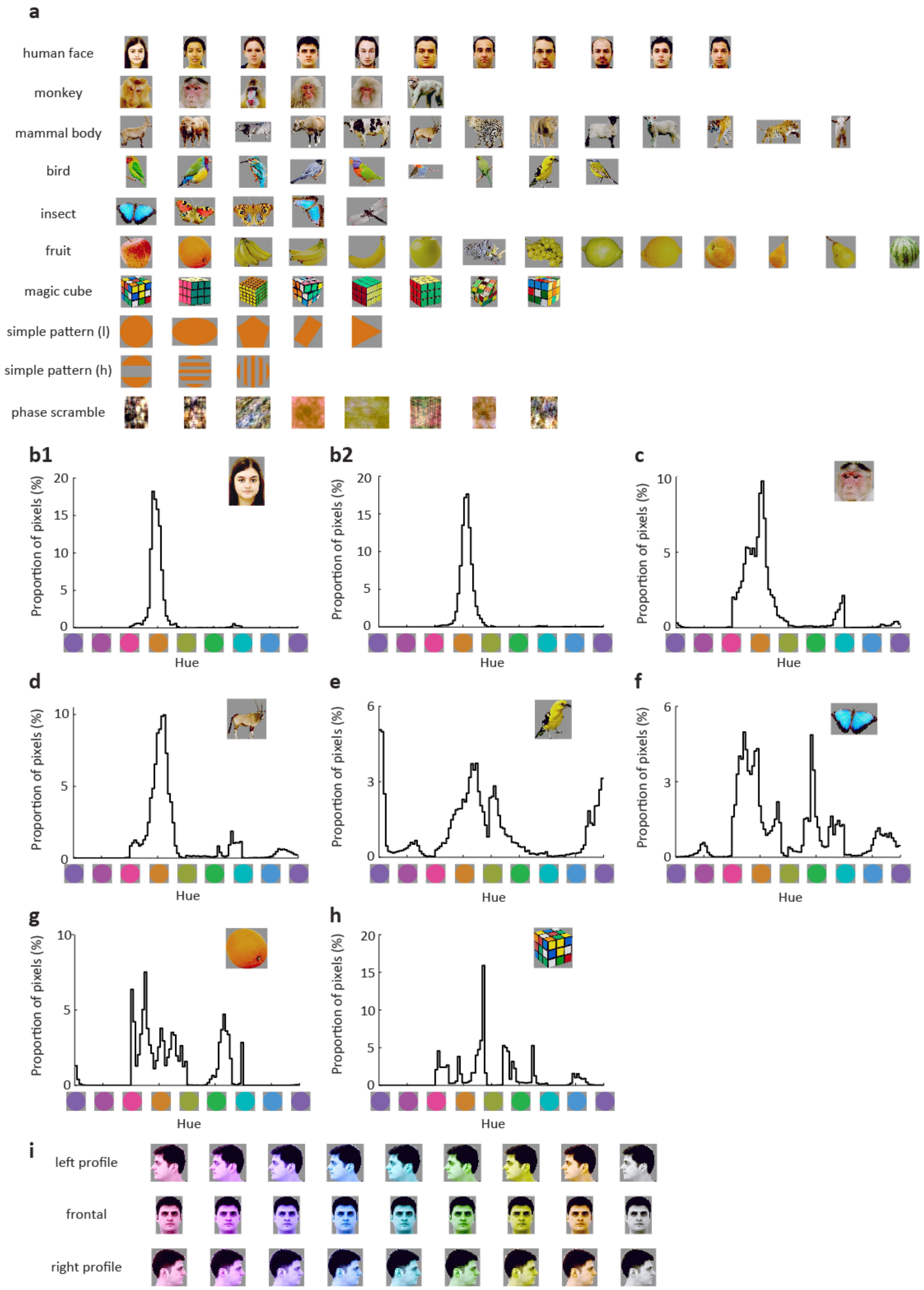
a
human face
monkey
mammal body
bird
insect
fruit
magic cube
simple pattern (l)
simple pattern (h)
phase scramble
b1
b2
c
d
e
f
g
h
Proportion of pixels (%)
Hue
i
left profile
frontal
right profile

Supplementary Figure 2. Stimuli.

a, Original 82 images. Each image was normalized to have the same mean luminance as the background. The last category contains eight phase scrambled images of two human faces, one mammal body, one bird, two fruits, one magic cube and one simple pattern (for details see Methods). **b-h,** Hue distribution of the images. **b1,** For each pixel of a human face image (inset), the angle of its hue was computed. Ambiguous pixels (defined as the 25% of pixels with the smallest distance to "white") were excluded, and the distribution of hue angles of the remaining 75% pixels is plotted. **b2**, average distribution of all 11 human faces. **c-h,** same as b2, but for 6 other categories. **i,** Similar to human faces in the main stimulus set (a), but with left and right profile images of the same identity added.

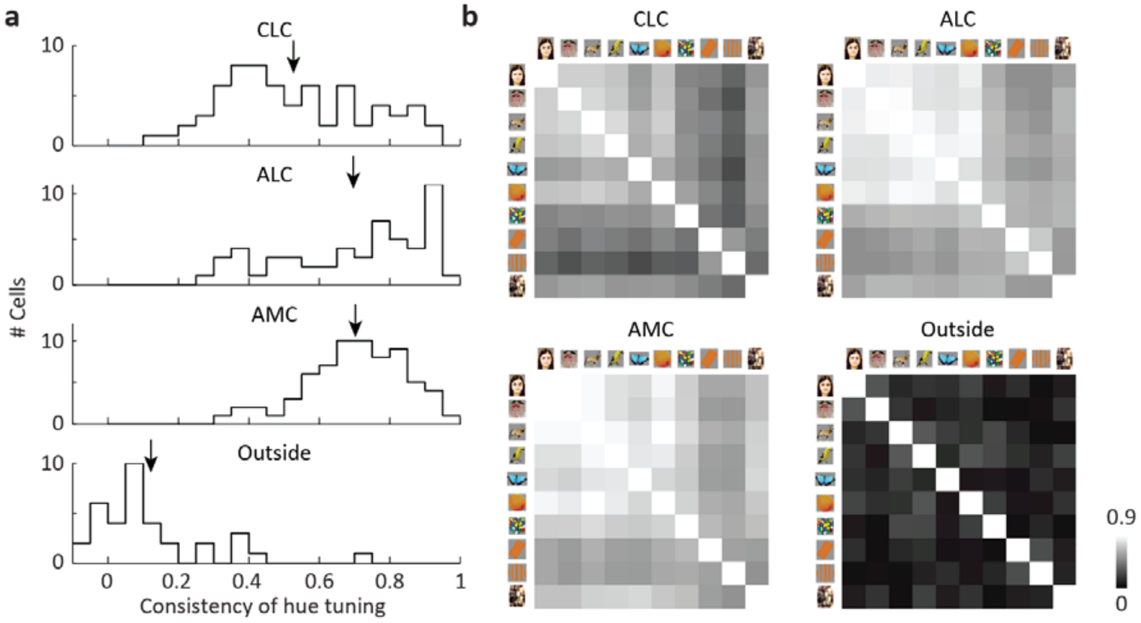

Supplementary Figure 3. Comparison of hue tuning consistency across targeted regions.

a, Correlation of hue tuning across eight hues between different categories was averaged for each cell to define the hue tuning consistency of the cell. Distributions of hue tuning consistency are plotted for three patches and outside color patches separately. Arrows indicate population average (=0.526 for CLC, 0.696 for ALC, 0.702 for AMC, and 0.119 for outside color patches). **b,** Correlation between responses to each pair of categories, averaged across all cells from each patch or outside color patches separately; each square represents average correlation between two 8-d vectors. In all three color patches, the correlation between any pair of the three categories with mammal images (including human faces, primate faces or mammal bodies) was higher than 0.74.

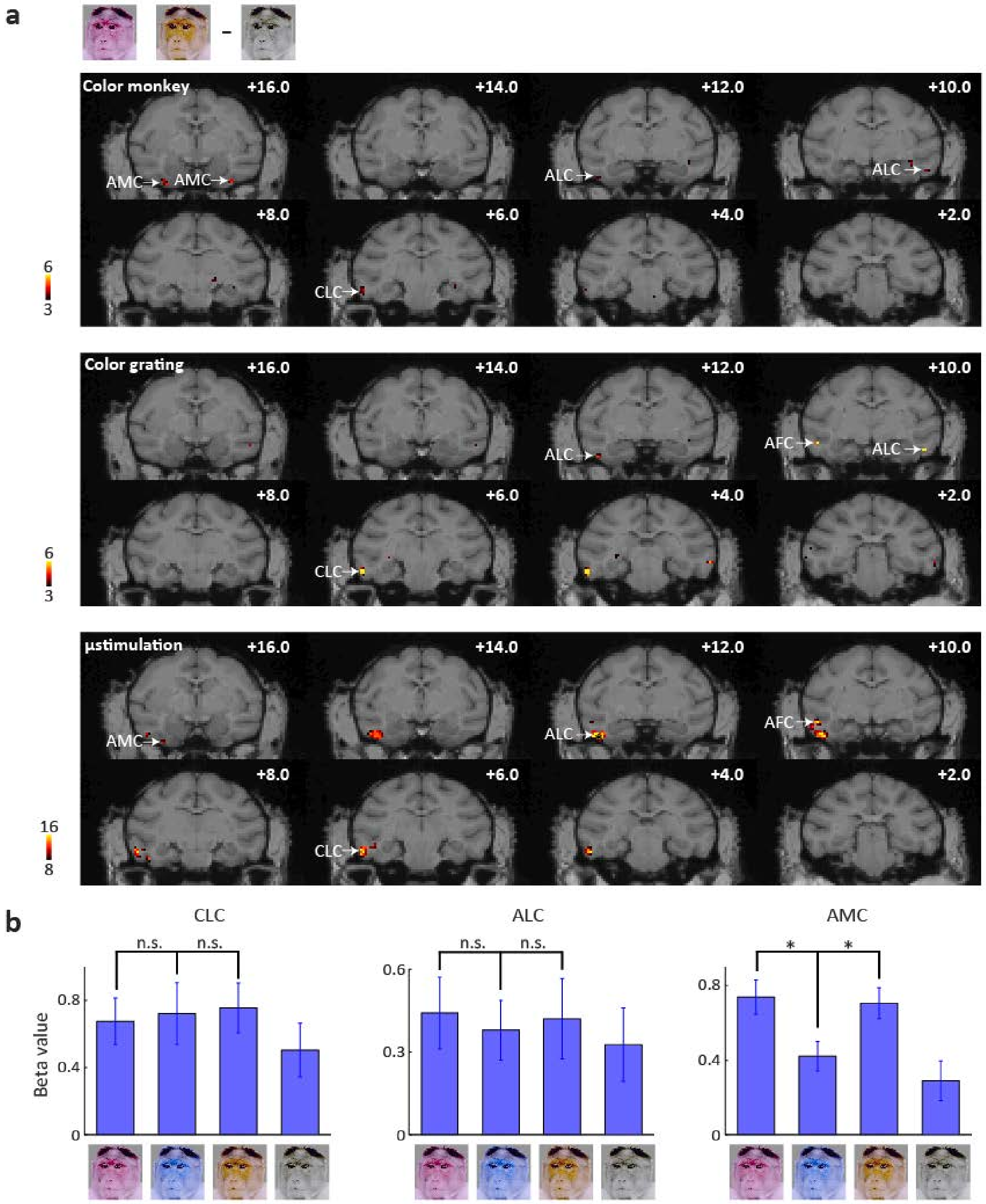

Supplementary Figure 4. fMRI reveals bias towards red/yellow monkey faces in AMC but not in CLC/ALC.

a, Comparison between color patches identified by colored monkey faces (top) and those by color gratings (middle) or microstimulation (bottom). The same convention as Fig. 1d. Using monkey faces clearly identified AMC in both hemispheres, with the left one overlapping with AMC located with microstimulation. Furthermore, CLC on the left and ALC on both hemispheres were identified, although the activation in these two patches was weaker than that by color gratings. **b,** Beta values for four stimulus conditions (red, blue, yellow and gray monkey faces) in CLC, ALC localized with color grating and AMC localized with microstimulation on the left hemisphere. Only AMC showed clear preference for yellow/red faces over blue faces. Error bars represent s.e.m. Student's t-test was used to determine statistical significance between conditions (*=$p<0.05$; CLC: $t(38)= -0.5234$ and 0.3754, $p=0.60$ and 0.71; ALC: $t(38)=0.9817$ and 0.6069, $p=0.33$ and 0.55; AMC: $t(38)= 2.6823$ and 2.5439, $p=0.011$ and 0.015).

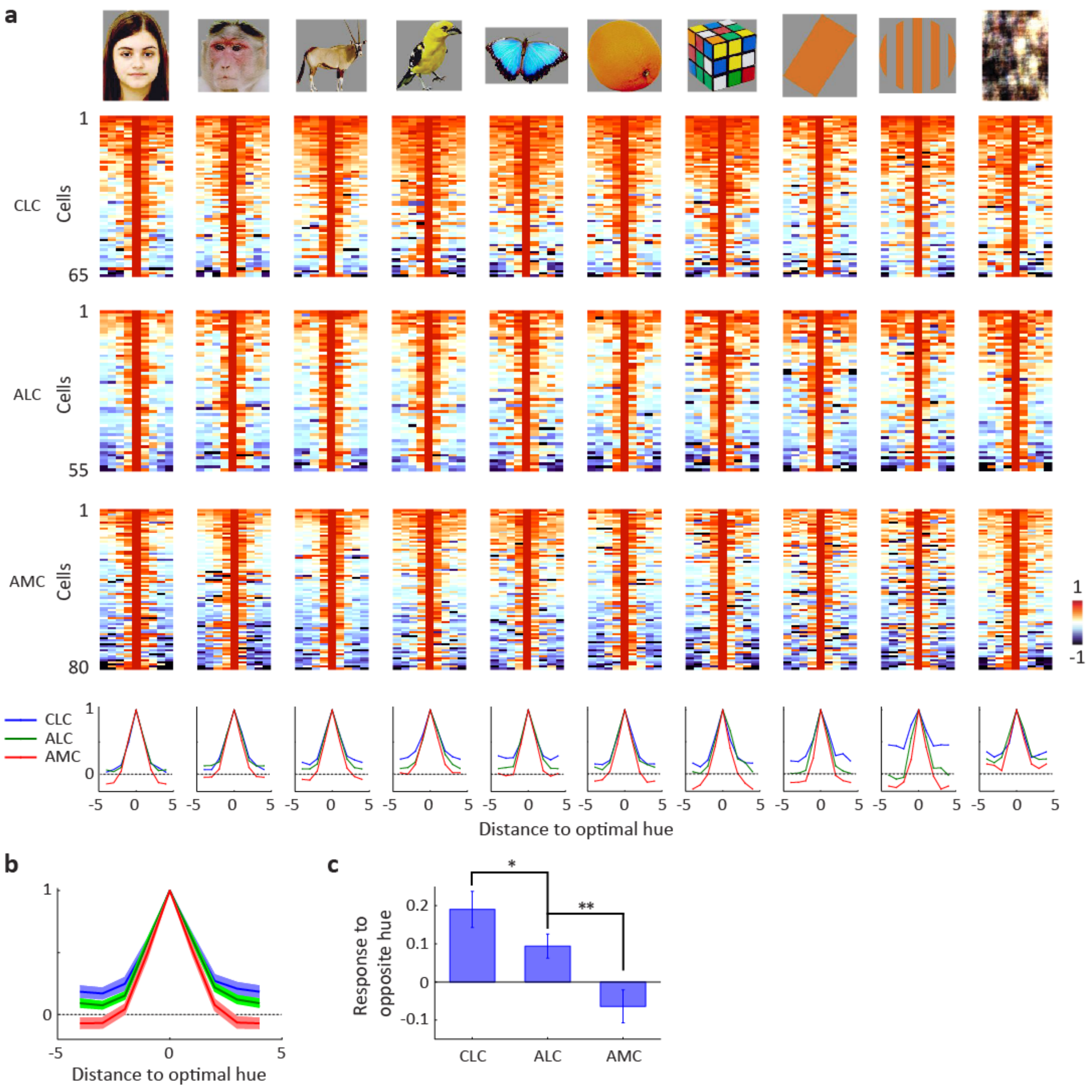

Supplementary Figure 5. Hue tuning is gradually sharpened from CLC to ALC to AMC.

a, Responses to 8 different hues were re-centered according to the optimal hue, and compared across three patches. Responses to gray stimuli with zero contrast were used to define a baseline and were subtracted from responses to other images. Average tuning across neurons in each patch are shown below. In nearly all cases, AMC shows sharper tuning than CLC, with ALC in the middle. **b,** Hue tuning like in (a) averaged across all 82 images for three patches. Shaded regions indicate s.d. of 2000 iterations of bootstrapping. **c,** A Gaussian function was fitted to the tuning function for each bootstrap sample. Mean and s.d. of the ratio between the opposite hue (distance to optimal hue=4) and optimal hue were plotted. Statistical significance were determined between neighboring patches (*=p<0.05, **=p<0.01).

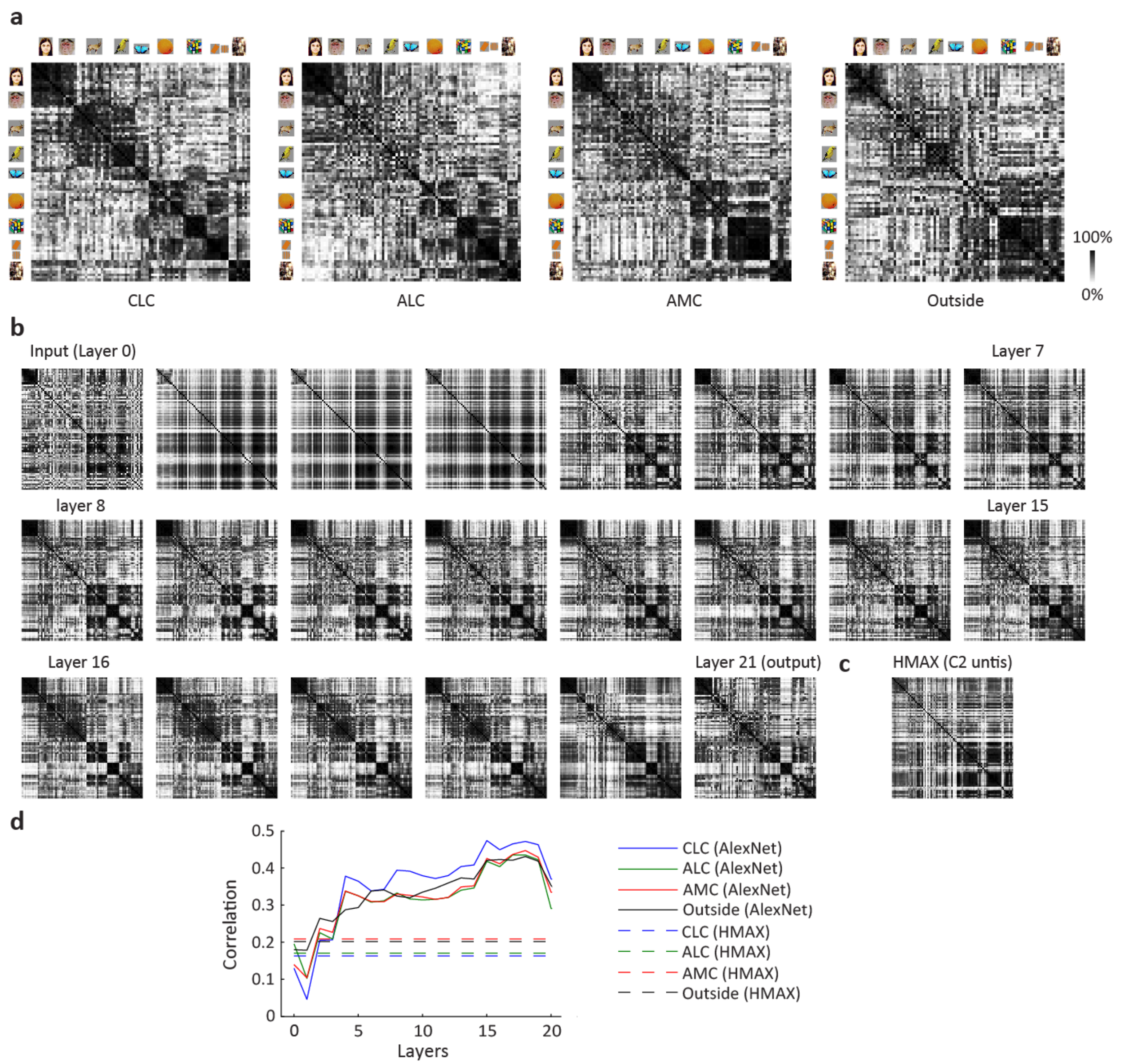

Supplementary Figure 6. Comparison of shape representation between color patches and neural network models.

a, Population similarity matrices computed from responses to object shapes averaged across hues in three color patches and IT cells outside the color patches. The matrices were rank-normalized (white=0th percentile, black=100th percentile). **b,** 82 grayscale images were loaded into a deep neural network pre-trained to identify a thousand different objects: AlexNet (Krizhevsky et al., 2012). Population activity of units in each layer was used to compute a similarity matrix. AlexNet becomes fully connected at layer 16. **c**, same as (b), but for a different model implementing invariant object representation: HMAX model (Riesenhuber and Poggio, 1999). C2 units of HMAX model were shown to nicely reproduce responses of V4 neurons (Cadieu et al., 2007). **d,** Correlation of similarity matrices between each patch and each layer

of AlexNet. Dashed lines represent correlation with C2 units of HMAX model. Similarity matrices from CLC, ALC and AMC best matched layer 15,17 and 18 of AlexNet, and IT cells outside color patches best matched layer 18.

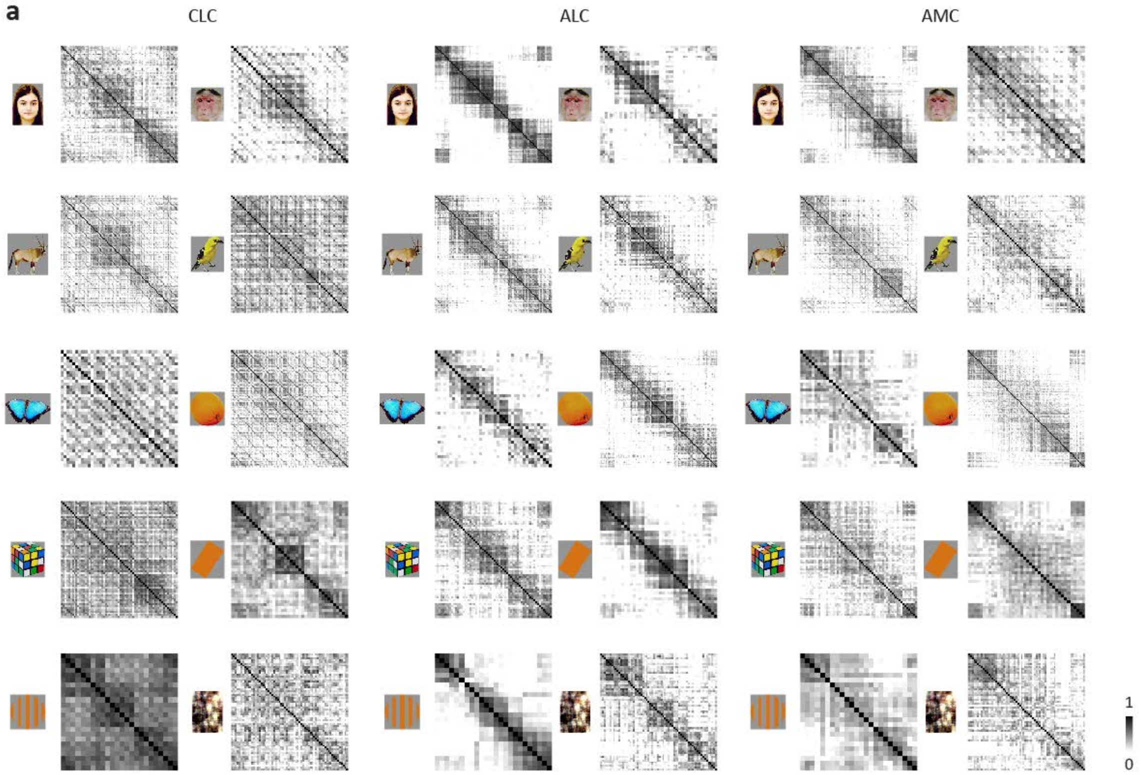

Supplementary Figure 7. Co-representation of hue and object identity for 10 different categories.

Same as Fig. 5b, but for all 10 categories.
